# An Exceptionally Rare Cause of Refractory Gastrointestinal Bleed: Choriocarcinoma Syndrome

**DOI:** 10.7759/cureus.14599

**Published:** 2021-04-20

**Authors:** Jennifer Yoon, Steve Hu, Jessica Farrell, Kandarp K Shah, Jaya Krishna Chintanaboina

**Affiliations:** 1 Internal Medicine, University of California San Francisco Fresno, Fresno, USA; 2 Gastroenterology and Hepatology, University of California San Francisco Fresno, Fresno, USA; 3 Pathology, Community Medical Center, Fresno, USA; 4 Gastroenterology and Hepatology, Community Medical Center, Fresno, USA

**Keywords:** refractory gastrointestinal bleed, small intestinal metastasis, choriocarcinoma, choriocarcinoma syndrome, gastrointestinal bleeding, gastrointestinal neoplasms

## Abstract

Testicular choriocarcinomas comprise less than 1% of all testicular tumors and are often highly vascular with early hematogenous metastasis. Choriocarcinoma syndrome (CS) is a rare entity distinguished by diffuse tumor burden and often fatal bleeding from metastatic sites. Most reported cases describe pulmonary hemorrhage secondary to initiation of chemotherapy. We present a fatal case of a young, previously healthy male with overwhelming gastrointestinal bleeding as the presenting sign of CS. Our case demonstrates that CS should be considered in the differential diagnosis for refractory anemia due to gastrointestinal hemorrhage in a young male with a testicular mass.

## Introduction

Testicular choriocarcinoma is a rare, highly aggressive subtype of non-seminomatous germ cell tumors and comprises approximately 0.2% of all testicular tumors [1]. Hematogenous metastases usually occur early in the disease course, and therefore, most patients typically present in an advanced stage with expected poor prognosis.

Choriocarcinoma syndrome (CS) is an exceedingly rare and often fatal manifestation of choriocarcinoma and is likely a reflection of massive tumor burden [2]. The majority of cases present with pulmonary hemorrhage; however, there are rare cases that demonstrate spontaneous hemorrhage from the gastrointestinal tract (GIT), liver, and brain [3,4]. CS usually develops after initiation of chemotherapy and is rarely the initial feature of choriocarcinoma [5]. Therefore, there is a paucity of literature to detail gastrointestinal hemorrhage as the presenting symptom of CS [6].

Here, we describe an uncommon case of a young male who presented with overt gastrointestinal bleeding (GIB) as the solitary sign of CS.

## Case presentation

A 32-year-old previously healthy male was transferred from an outside facility after presenting with several weeks of progressively worsening fatigue, abdominal pain, non-bloody vomiting, and melena. Prior to transfer, he received two units of packed red blood cells due to a low initial hemoglobin of 4 g/dL (14-18 g/dL). He was hemodynamically stable. On physical examination, his abdomen was soft and lax. Admission labs were notable for a post-transfusion hemoglobin of 5.3 g/dL. The chemistry, coagulation, and liver panels were otherwise unremarkable.

Esophagogastroduodenoscopy revealed a non-bleeding arteriovenous malformation (AVM) in the gastric body which was treated with hemostatic clips. Colonoscopy was unremarkable.

**Figure 1:**
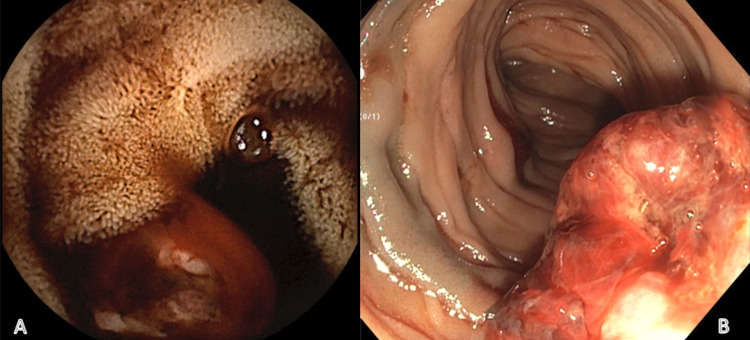
(A) Active hemorrhage from jejunal mass visualized by video capsule endoscopy. (B) Jejunal mass visualized by small bowel enteroscopy.

**Figure 2:**
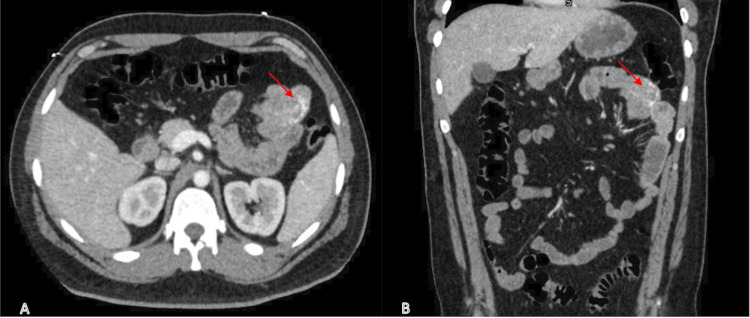
(A) Axial and (B) coronal contrast-enhanced CT enterography demonstrating an area of serpiginous enhancement in the jejunum (left upper quadrant) suggestive of active hemorrhage. CT, computed tomography

**Figure 3:**
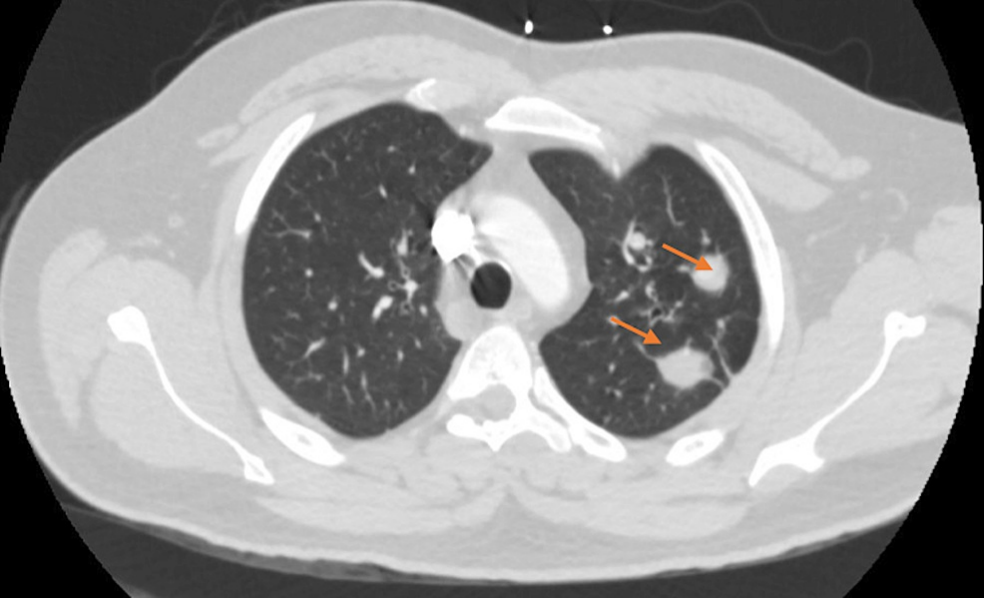
Axial view of CT angiogram pulmonary demonstrating two large, closely clustered pulmonary nodules with large serpentine enhancing vessels within the posterior lateral left upper lobe. CT, computed tomography

A video capsule endoscopy showed a proximal jejunal lesion. Push enteroscopy revealed a 1.5 cm bleeding mass in the proximal jejunum, which was biopsied (Figure 1). A computed tomography (CT) enterography revealed an area of serpiginous enhancement within the jejunum in the left upper quadrant concerning for active hemorrhage (Figure 2). A CT of the chest revealed innumerable bilateral pulmonary nodules suspicious for extensive pulmonary AVMs (Figure 3).

**Figure 4:**
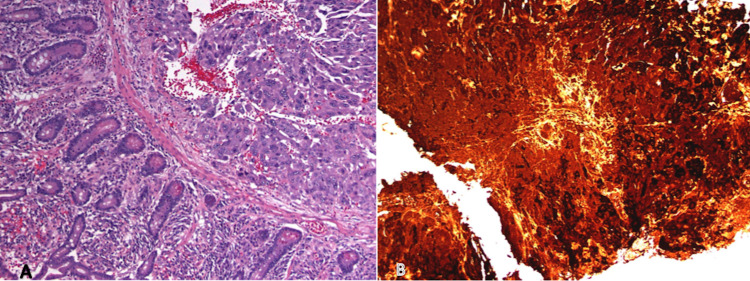
(A) Representative slide from resected jejunal tumor demonstrating choriocarcinoma with adjacent normal jejunum (H&E stained, 40× magnification). (B) Immunohistochemistry staining with beta-hCG of jejunal tumor confirming choriocarcinoma (beta-hCG, 40× magnification). hCG, human chorionic gonadotropin

Due to persistent bleeding from the mass, the patient underwent emergent exploratory laparotomy with resection of 50 cm of the proximal jejunum with primary two-layer, hand sewn jejuno-jejunal anastamosis. The excised surgical specimen of the jejunal mass confirmed metastatic choriocarcinoma (Figure 4).

Amidst the workup, the patient reported left testicular pain and had visible scrotal swelling. Testicular ultrasound revealed a 5 × 3 cm left intra-testicular mass and beta-human chorionic gonadotropin (hCG) was over 200,000 IU/L. He was considered a poor surgical candidate for orchiectomy given his overall poor clinical status with recent exploratory laparotomy. He was started on chemotherapy with cisplatin and concurrent reduced doses of ifosfamide and etoposide. Unfortunately, he developed complications related to septic shock with multiorgan failure and expired on day 36 of hospital admission.

## Discussion

CS is an extremely rare clinical condition associated with high morbidity and mortality that was first described in 1984 [7]. CS is described as a unique presentation in patients that demonstrates an exceptionally aggressive choriocarcinoma subtype, often displaying widespread lung metastases and high beta-hCG levels (>50,000 IU/L) [8]. The distinguishing feature for this syndrome involves massive hemorrhage from metastatic sites due to a large tumor burden which quickly outgrows its vascular supply [9]. Reports on the metastatic behavior of testicular choriocarcinoma in the GIT are limited; malignancies with GIT metastases usually originate from the breast, lung, and melanoma [10].

CS often manifests as massive tumor lysis secondary to initiation of chemotherapy. Rarely, it can present as primary spontaneous hemorrhage in metastatic choriocarcinoma [11,12]. Most cases present with alveolar or cerebral hemorrhage. Our patient experienced severe, recalcitrant GIB despite also having widespread pulmonary metastases and beta-hCG over 200,000 IU/L. Significant GIB has been reported in up to 5% cases of metastatic choriocarcinoma, including involvement of the stomach, small intestine, and colon [13,14]. Despite the lack of obvious alveolar hemorrhage, the diagnosis of CS should not be overlooked in patients with primarily GIB and additional features of an aggressive choriocarcinoma.

Definitive treatment options for choriocarcinoma-related GIB are rather limited. Hemorrhage is cited as the cause of death for 44% of patients with testicular choriocarcinoma [15]. The 1997 International Germ Cell Cancer Collaborative Group and the 2018 European Society for Medical Oncology (ESMO) consensus conference denote patients with pure choriocarcinoma and high hCG (>50,000 IU/L) as having “poor” prognosis with often fatal hemorrhage despite chemotherapy [16,17]. The treatment approach to GIB in CS is similar to that of a typical gastrointestinal hemorrhage. Timely resuscitation with blood products followed by targeted hemostatic options consisting of endoscopic interventions, angiogram with embolization, and surgical interventions are all plausible treatment options [9].

Nonetheless, targeted treatment of choriocarcinoma is paramount to attaining control of the rapidly growing metastatic burden [14]. Ideally, patients who are found to have a solid testicular mass should undergo early radical orchiectomy which is both a diagnostic and therapeutic intervention [8]. Data are sparse on the treatment for metastatic choriocarcinoma with CS and is individualized based on disease burden. ESMO guidelines recommend that those with metastatic pure choriocarcinoma and high hCG should receive induction therapy with full-dose cisplatin and etoposide [16]. Due to adverse pulmonary effects, bleomycin (or ifosfamide) is deferred until the patient is more stable [18]. Orchiectomy can be considered several weeks later if patients remain stable after initiation of chemotherapy [16]. As there is high risk of complications such as CS after chemotherapy, early referral to a high-volume center with experience in treatment of advanced germ-cell tumors should be strongly considered [4]. Overall, prognosis for patients with GIT involvement is extremely poor; survival rate is 30% and typical survival time has been reported to be from several days to up to 15 months [19,20].

## Conclusions

We report a rare case of CS with jejunal metastasis causing overt GIB. Diagnosis and management can be difficult secondary to its nonspecific presentation and often delayed diagnosis at late stages of the disease. Early recognition and treatment with chemotherapy is a mainstay; however, most patients still are at high risk of fatal complications. Our case highlights the comprehensive workup for obscure overt GIB and the need to consider metastatic choriocarcinoma in the differential diagnosis in a young male with GIB and testicular mass.
